# Evaluation of Network Design and Solutions of Fisheye Camera Calibration for 3D Reconstruction

**DOI:** 10.3390/s25061789

**Published:** 2025-03-13

**Authors:** Sina Rezaei, Hossein Arefi

**Affiliations:** i3mainz—Institute for Spatial Information and Surveying Technology, School of Technology, Mainz University of Applied Sciences, D-55118 Mainz, Germany; rezaei.sina@hs-mainz.de

**Keywords:** camera calibration, network design, 3D reconstruction, fisheye lens, spherical camera, accuracy

## Abstract

The evolution of photogrammetry has been significantly influenced by advancements in camera technology, particularly the emergence of spherical cameras. These devices offer extensive photographic coverage and are increasingly utilised in many photogrammetry applications due to their significant user-friendly configuration, especially in their low-cost versions. Despite their advantages, these cameras are subject to high image distortion. This necessitates specialised calibration solutions related to fisheye images, which represent the primary geometry of the raw files. This paper evaluates fisheye calibration processes for the effective utilisation of low-cost spherical cameras, for the purpose of 3D reconstruction and the verification of geometric stability. Calibration optical parameters include focal length, pixel positions, and distortion coefficients. Emphasis was placed on the evaluation of solutions for camera calibration, calibration network design, and the assessment of software or toolboxes that support the correspondent geometry and calibration for processing. The efficiency in accuracy, correctness, computational time, and stability parameters was assessed with the influence of calibration parameters based on the accuracy of the 3D reconstruction. The assessment was conducted using a previous case study of graffiti on an underpass in Wiesbaden, Germany. The robust calibration solution is a two-step calibration process, including a pre-calibration stage and the consideration of the best possible network design. Fisheye undistortion was performed using OpenCV, and finally, calibration parameters were optimized with self-calibration through bundle adjustment to achieve both calibration parameters and 3D reconstruction using Agisoft Metashape software. In comparison to 3D calibration, self-calibration, and a pre-calibration strategy, the two-step calibration process has demonstrated an average improvement of 2826 points in the 3D sparse point cloud and a 0.22 m decrease in the re-projection error value derived from the front lens images of two individual spherical cameras. The accuracy and correctness of the 3D point cloud and the statistical analysis of parameters in the two-step calibration solution are presented as a result of the quality assessment of this paper and in comparison with the 3D point cloud produced by a laser scanner.

## 1. Introduction

The camera is recognised as the primary tool in photogrammetry for measurement and information generation. This optical instrument, depending on its geometric structure and sensor type, can provide 2D information, semantic information, metric products, and precise practical data. Recently, the use of spherical cameras has gained attention in various projects [1]. Their extensive photographic coverage and ability to obtain necessary data for the production of augmented reality, digital twins [2], and 3D reconstructions have made them increasingly viable [3,4]. These cameras are generally available in professional and low-cost models. The low-cost models, with their simpler configurations, offer users easier operation and faster capture, which are often preferred for their accessibility and affordability [5,6]. However, several factors prevent the use of this type of camera in most photogrammetric studies. One of them is the high distortion of the images produced by these cameras, which requires a calibration tailored to the geometry of fisheye images. This problem has been studied extensively in photogrammetry to determine the optical image parameters [4,7], which mainly include focal length (f), the pixel position of the image’s geometric centre (cx, cy), radial distortion parameters (K1, K2, K3, K4), tangential distortion parameters (p1, p2), and the non-orthogonality of physical axes of the camera lens (b1, b2) [8]. Camera calibration is essential for accurate image processing to determine positions from photographs, create 3D reconstructions, and extract any metric information from image-based products [9]. To accurately determine the relationship between image space and object space, a projection model is required [8].

Recent advancements in calibration methods focus on achieving high accuracy in 3D reconstruction tasks by addressing the complexities of spherical images [3]. Moreover, the development of self-calibration methods for fisheye lenses has provided insights into optimising calibration processes under diverse conditions [8]. These methods allow for adaptive approaches that cater to specific use cases, such as the digitisation of cultural heritage structures and urban environments, where cost-effective solutions are essential [5,6]. The accuracy of the calibration methods and the design of the camera network during calibration are critical in evaluating the calibration itself [10]. Despite using the latest orientation method with least squares adjustment for 3D reconstruction, which leads to the highest accuracy and reliability among other mathematical approaches, the lack of an accurate camera calibration and the weak network design geometry for calibration cause poor results in the accuracy metric. Since the least squares method includes nonlinear equations, sufficient initial values need to be considered in the following orientation steps of 3D point cloud generation [11]. Therefore, in order to improve the assessment of the quality and accuracy of the calibration of spherical cameras with fisheye lenses, studying the impact of calibration parameters on the production of 3D products, such as 3D point clouds, can serve as a clear indicator of the importance of this aspect in the quality of processing outputs. This quality analysis of the camera calibration is performed according to the known criteria, such as the stability and accuracy of the calibration parameters, re-projection error, aggregation of the number of tie points in the constant value of the re-projection error, registration accuracy of ground control points, and correctness of the pose estimation for the camera position. For the purpose of affordability and easy configuration for non-professional users, professional spherical cameras such as the Teledyne FLIR Ladybug, Professional 360 Panono, Insta360 Pro2, and Weiss AG Civetta [3,12] are excluded from the considered spherical cameras.

The calibration of fisheye lenses presents challenges due to their inherent distortions, which require specialised models and techniques for effective compensation. Studies have demonstrated the potential of low-cost fisheye cameras for close-range photogrammetry, particularly for indoor dimensional measurements, emphasising the balance between affordability and accuracy [1]. Similarly, fisheye cameras have been effectively used for creating digital twins of historic structures and enabling the detailed monitoring and documentation of deterioration [2]. There are several approaches for fisheye camera calibration, which can be divided into two main parts. The first part involves using a relative orientation constraint to optimise camera calibration [13], which is known as self-calibration, and the second part uses a planar target or pattern to calculate stable calibration parameters, also known as pre-calibration. In recent years, thanks to the combination and improvement of the current state of these two calibration solutions, other fisheye camera calibrations, including two-step calibration [14] and 3D calibration, have emerged as alternative solutions to improve the geometric structure of the resulting 3D point cloud and reduce the distortion of the images. The two-step camera calibration solution is defined by removing the influence of distortion on the images [15], while the 3D calibration concept follows the control point constraint and is influenced by the bundle approach. In fact, these two alternatives use the basic self-calibration and pre-calibration calculation methods [13,15].

The confined space and limited lighting conditions of the underpass presented significant challenges for optical imagery, particularly in close-range photogrammetry network design and feature capture. These difficulties were most pronounced in the middle section, where minimal lighting and the presence of stairs further restricted space. The use of a spherical camera introduced fisheye images as raw data, increasing distortion and reducing geometric consistency, which in turn affected the accuracy and complexity of relative orientation and camera calibration. Achieving high accuracy with fisheye lenses required an optimized camera network design, as their wide field of view complicated chessboard-based calibration and the selection of camera station configurations for both calibration and 3D reconstruction. To address these challenges, we tested two different spherical cameras, explored multiple calibration approaches using two separate software solutions, and carefully designed the photogrammetry workflow. By evaluating both relative and absolute solutions through a controlled dataset and a review of previous studies, we identified the most effective camera calibration approach, which holds potential for further refinement in future research.

This research utilizes five primary fisheye camera calibration strategies (pre-calibration, self-calibration, two-step calibration (solution 1 and 2), and 3D calibration) to examine the efficiency and accuracy of the results. The objective is to illustrate the most suitable calibration option. This study also involves the analysis and assessment of software and any factors that impact the final calibration results. Additionally, it utilizes calibration parameters in 3D reconstruction to practically demonstrate the effects of each calibration strategy on the geometry consistency and accuracy of 3D point clouds.

This paper is organized into five sections. The introduction provides a comprehensive overview of the fundamental concepts related to fisheye camera calibration, along with its background and relevant fields. This serves as a foundation for understanding the subsequent sections. Section 2 (Workflow of Research) defines the workflow and framework of this study, introducing the case study and its key features. This section establishes the structural approach adopted in this research. Section 3 focuses on the experimental process (Experiments), including data acquisition and data organization. This ensures a clear understanding of the research dataset before delving into the methodological aspects. Section 4, Methods, details the procedures, techniques, and solutions employed in this study. It presents a step-by-step explanation of the approach used for fisheye camera calibration and its application. Finally, the last section provides a thorough evaluation of the calibration results. The results are analysed from both relative and absolute perspectives, offering the assessment of the fisheye camera calibration process and its effectiveness in 3D reconstruction.

## 2. Workflow of Research

**Figure 1:**
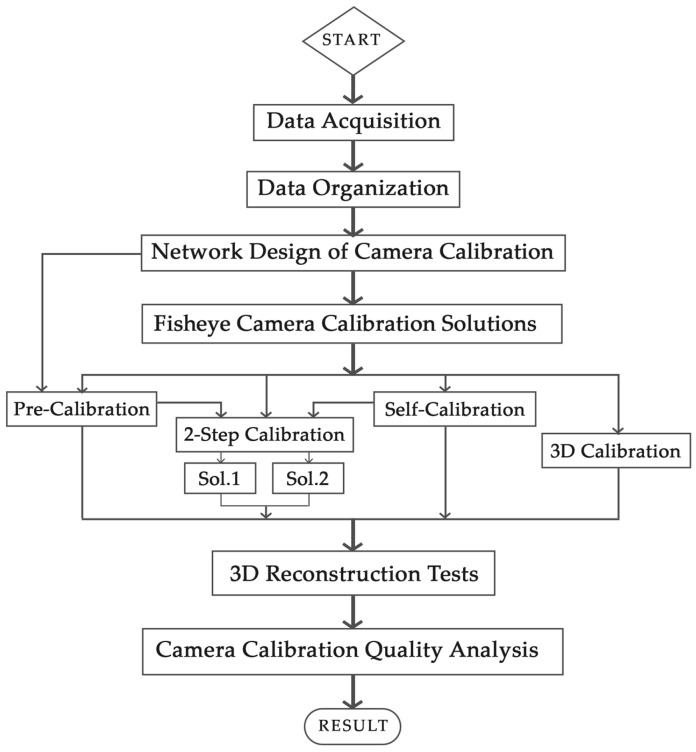
The workflow of this research.

The overall framework of this research is based on the demonstration of the workflow, including several well-known tests from recent studies on the calibration of fisheye images (Figure 1) [12,14,15,16]. In Section 3 and Section 4, the details of the procedure will be explained, and, in the final section, they are evaluated both in absolute and relative terms, and the impact of the estimated calibration parameters on the 3D reconstruction of a structure is also assessed. As a prerequisite, primary data are collected from the structure of interest for 3D reconstruction (using a GNSS receiver for control point measurement and a laser scanner as the reference dataset) and calibration testing (in the 3D calibration solution, control points are measured by the total station). Initial image processing is performed on the raw data in the data organization part to prepare the images for calibration tests and the other datasets for 3D reconstruction. Subsequently, the impact of the calibration network design on the accuracy of the calibration parameters is first examined through a series of tests by using the spherical cameras (Insta360 X2 and X3—Arashi Vision Inc., Shenzhen, China), with the best result being identified as the output. At this point, the pre-calibration test is accomplished. The upcoming calibration tests include 3D calibration, self-calibration, pre-calibration, and two-step calibration tests, which are performed using two solutions. The calibration parameters obtained from each test are then used in the 3D reconstruction of the case study during the relative orientation and bundle adjustment processes. The quality analysis section evaluates the accuracy and reliability of the calibration parameters in comparison with the results of the self-calibration; as a reference, the results obtained in a previous work are used [17]. In this section, the quality of the resulting 3D point cloud is also examined by two individual spherical cameras, in comparison to a reference laser scanner 3D point cloud.

### Case Study

**Figure 2:**
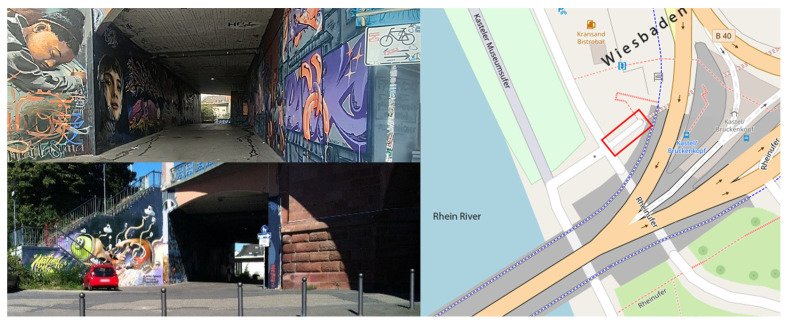
Case study of research; Theodor-Heuss Bridge in Wiesbaden, Germany. The red border indicates the location of the underpass.

In the case of the analysis of calibration parameter results in the relative orientation and 3D point cloud, the case study for the 3D reconstruction step is the graffiti-covered underpass under the Theodor-Heuss Bridge in Wiesbaden, Germany (Figure 2). This bridge was used in a previous work [17]. This bridge is an infrastructure link between the cities of Mainz and Wiesbaden, which has concrete walls decorated with various graphic artworks. This underpass is approximately 30 m long, 4.5 m high, and 6 m wide, and features a narrow arched design and sloped floor, making it a space-constrained structure that is challenging for 3D reconstruction. The light condition is not as strong at the two opening sides of the underpass, but it was sufficient to capture the image data type.

## 3. Experiments

### 3.1. Data Acquisition

As the main low-cost spherical cameras for this research, based on the previous work, Insta360 One X2 and Insta360 One X3 (Arashi Vision Inc., Shenzhen, China) were used to capture fisheye images for the main dataset. Two professional commercial software programs, Agisoft Metashape (version 2.1) and MATLAB (version 9.12, release: R2022a), with reliable frameworks for camera calibration, were used to perform calibration tests in the pre-calibration phase [18] and to validate the results obtained. OpenCV (vesion 4.8.0) was used to produce undistorted images from the calibration parameters and to separate raw images from the front and rear lenses while adjusting the image format for Metashape. Additional calibration tests and 3D reconstruction processing were conducted in Metashape.

**Figure 3:**
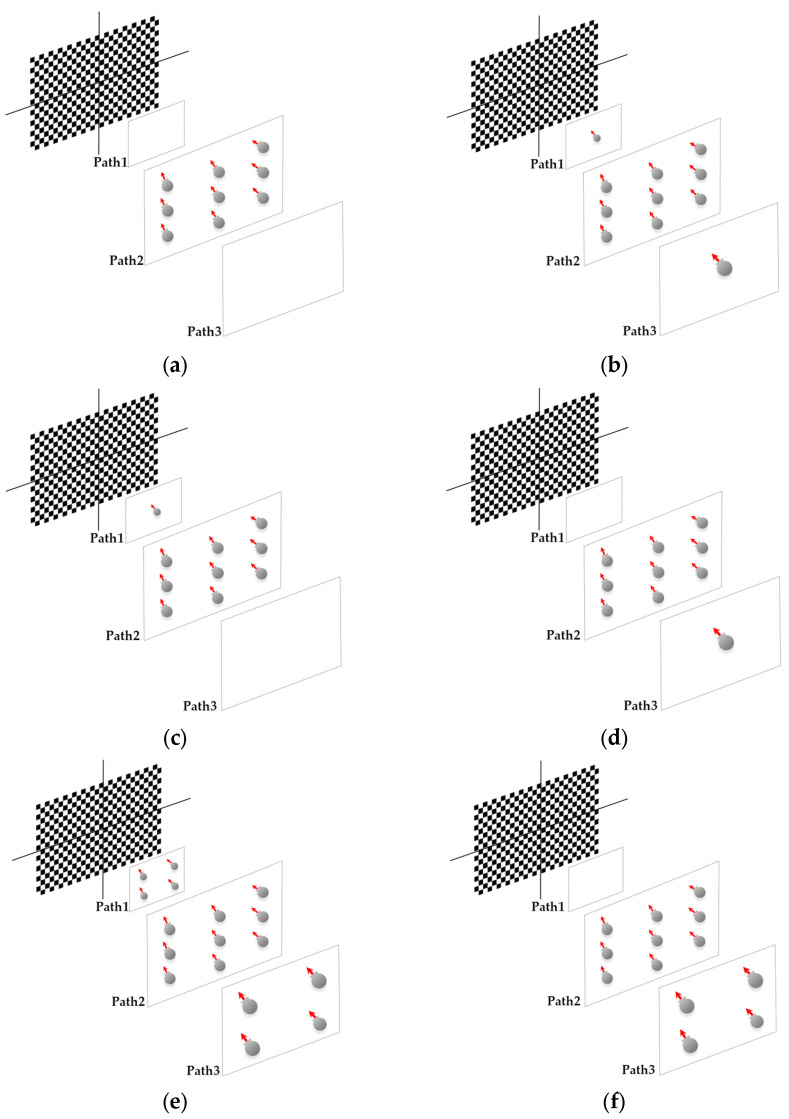

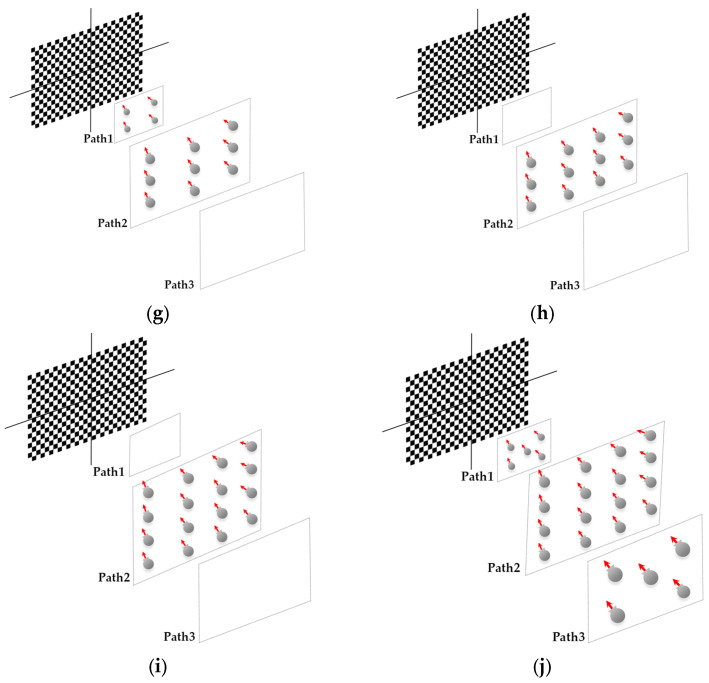
Network design tests configuration (each interval increases the distance of the camera from the chessboard): (**a**) 9 images in single path, (**b**) 11 images in 3 paths, (**c**) 10 images in 2 paths (the first and second paths), (**d**) 10 images in 2 paths (the second and third paths), (**e**) 17 images in 3 paths, (**f**) 13 images in 2 paths (the second and third paths), (**g**) 13 images in 2 paths (the first and second paths), (**h**) 12 images in single path, (**i**) 16 images in single path, and (**j**) 26 images in 3 paths.

**Figure 4:**
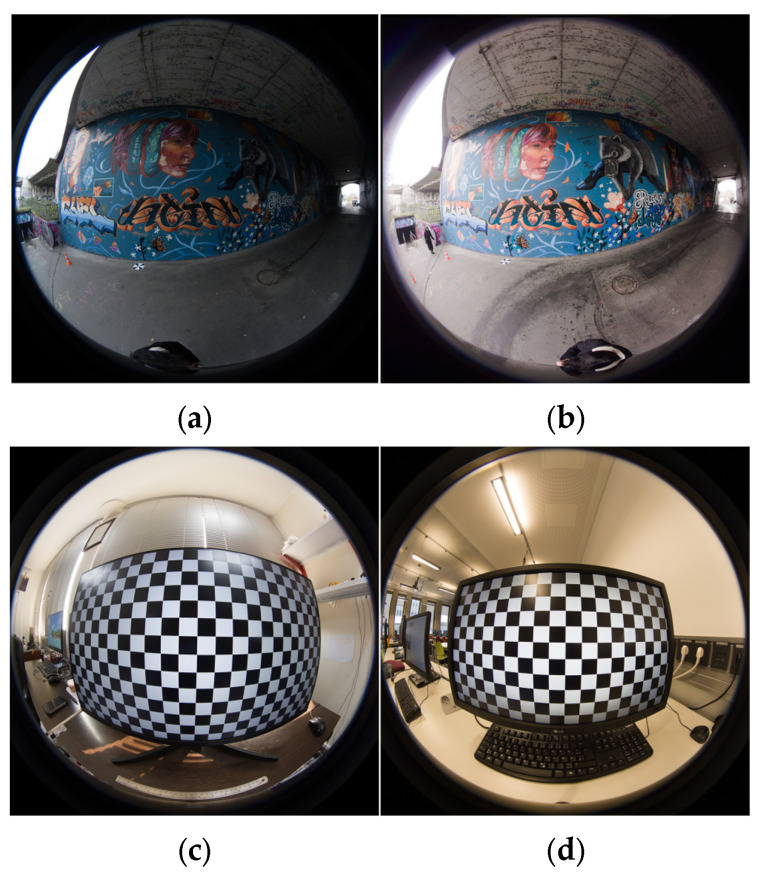
Sample front-side fisheye image using Insta360 One (**a**) X2 and (**b**) X3 from graffiti underpass, and sample front-side fisheye image using Insta360 One (**c**) X2 and (**d**) X3 from chessboard.

The image acquisition for each test was performed as follows. For the pre-calibration tests based on Figure 3, the 24 × 14 chessboard was visualized in the 27-inch monitor and according to the coverage area of the chessboard (Figure 4c,d). A total of 26 images were captured with three individual distances and orientations. Similar procedures were repeated for other calibration tests with their corresponding considerations according to Figure 3, except for the 3D calibration, where we captured 22 images from the photogrammetric 3D calibration targets field in two rows. For the control point definition in a local coordinate system, 25 points were measured with the total station as 3D control points. In the 3D reconstruction step, the previous work considerations for network design and data acquisition were assumed [17]. The camera height was set to 2.7 m. Images were acquired and positioned towards the walls (sideways mode) based on the geometry and corridor-shaped structure (Figure 4a,b). For the case of the reference source for the comparison of point clouds, the Leica RTC 360 laser scanner was used to capture the whole indoor area of the tunnel. The laser scanner point cloud was achieved through two stations of capture and using Cyclone Register 360 software (version 2021.1.2). For the purpose of comparison and reference source, the reconstructed walls of the tunnel were considered in the evaluation. For the registration for two capturing stations, Cyclone Register 360 software was used. There was a 73% total overlapping area specified between the two stations. More details about the LS capture are mentioned in Table 1. Also, for georeferencing the 3D point cloud of the tunnel, seven ground control points were observed in UTM coordinate system. The GNSS device with a Leica GNSS antenna was used for data correction services. The control points were located only on the ground (due to the urban restriction of mounting control points on the underpass wall).

**Figure 5:**
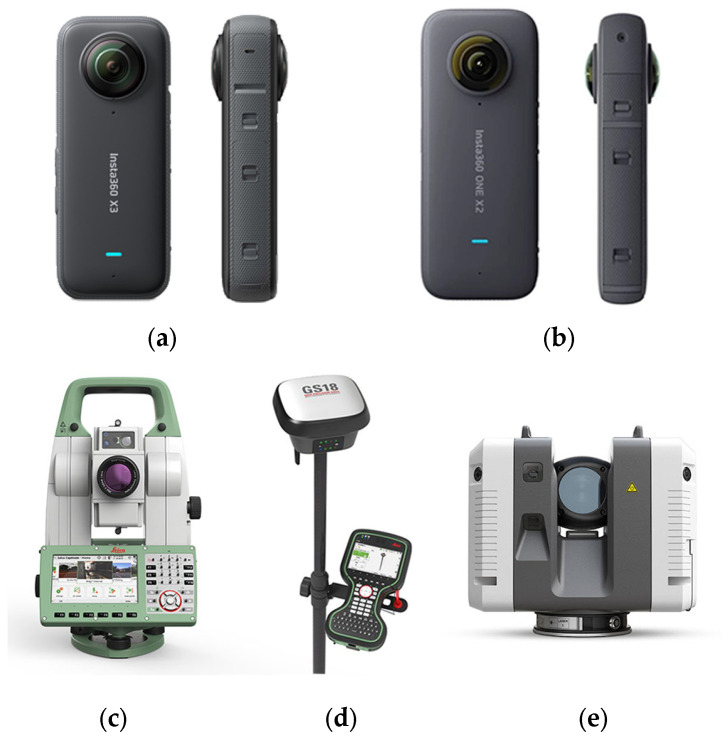
All sensors used in this study. (**a**) Insta360 One X3, one of the best available low-cost spherical cameras. (**b**) Insta360 One X2, one of the most optimized low-cost spherical cameras. (**c**) Total station and (**d**) GNSS receiver of Leica used for defining the network of control points. (**e**) Leica RTC 360 laser scanner, used as the reference sensor for point cloud quality assessment.

**Figure 6:**
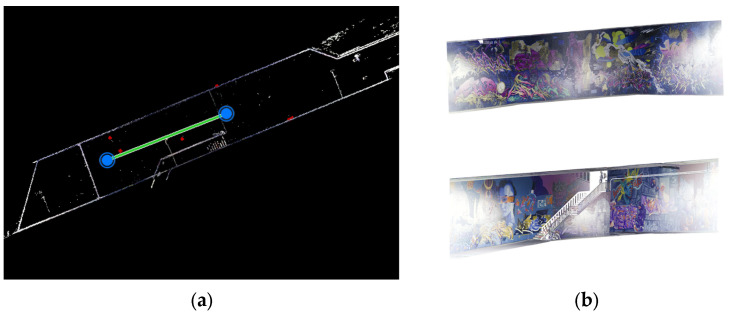
(**a**) Laser scanner 3D point cloud captured and organized into the right datatype format in Leica Cyclone software (E57 file into laz or las format, V2021.1.2) for further processing in Cloud Compare software (version 2.12 beta). (**b**) The walls of underpass, extracted from the entire point cloud, were used as reference data in Cloud Compare software in comparison to the spherical camera 3D point cloud.

For the purpose of control point measurements in the 3D calibration step, the Leica total station was used (Figure 5 and Figure 6).

### 3.2. Data Organization

**Figure 7:**
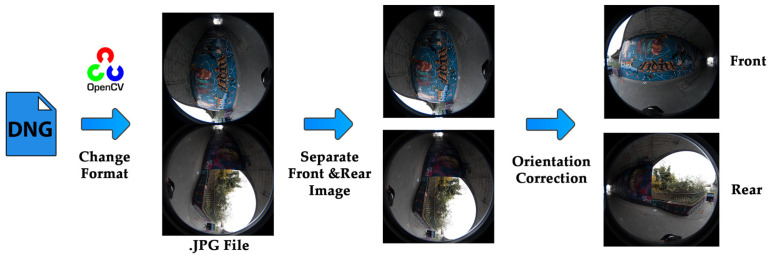
Workflow of data organization: change data format from DNG to JPG and detachment and orientation correction of front and rear lens images.

It is necessary to perform the initial dataset arrangement for further processing, including data type definition and fisheye image separation. While the fisheye images can be extracted directly from the raw file, the front and rear side images of the camera are separated from the raw file and rotated to a realistic viewpoint using OpenCV open-source computer vision libraries [19,20] in Python programming language (version 3.10). This was also repeated for the 3D reconstruction dataset using the sideways capture of the graffiti underpass (Figure 7).

## 4. Methods

### 4.1. Network Design of Camera Calibration

In addition to evaluating the accuracy and reliability of the parameters obtained from the pre-calibration method, the best network design with fisheye lenses is introduced into the calibration process. This is based on the fundamental principles of the network design or geometric configuration of cameras in photogrammetry, along with extensive research conducted in this field [7,11,21,22,23]. The goal is to show the various influences of different camera network configurations on the changes in the geometry of the resulting photogrammetric models and the quality of the 3D reconstruction result. Certain conditions are assumed, including the convergence of the acquired images, the need for adequate photographic coverage with most points of the checkerboard visible in the images (ideally all points should be visible), the depth variability of the acquired images to estimate the optimal focal length, and the checkerboard or target object covering most of the image space, especially when using fisheye lenses. Imaging at each station maintains constant camera geometric settings, such as focal length, to ensure the stability of internal orientation parameters. In this network design test, two parameters, depth variability and number of images, were evaluated to clarify the most optimal network configuration for camera locations. Accordingly, ten tests were performed using the pre-calibration method with the specifications listed (Figure 3). The evaluation of the tests was conducted through criteria such as re-projection error, accuracy and estimation of camera positions, as well as each of the calibration parameters. The depth variation was defined in three individual distances, including 20 cm, 60 cm, 100 cm between the centre of the chessboard screen and each camera position. These distances were defined based on the optimal covering area of the chessboard pattern in fisheye images, which were not too close and not so far, and all the squares of the pattern in the picture were observed.

### 4.2. Fisheye Camera Calibration Solutions

In general, the fisheye projection model cannot match the central perspective projection due to the special geometrical characteristics [24]. According to the research conducted on the fisheye lens characteristics [16,25,26] and based on the properties of the polydioptric spherical camera used in this research [3,27], the fisheye projection model follows the Equidistant projection. In order to fully characterize the distortions of the lens, the nonlinear Brown’s distortion model was combined with collinearity equations [16,28] (Equations (7) and (8)). The calibration procedure accomplishes the indirect estimation process of inner orientation parameters (IOPs), which include the focal length (f), normalized image coordinates (*X*_0_, *Y*_0_), a sample 3D position of a point (X, Y, Z), the principal point coordinates (*x*_0_, *y*_0_), in which the purpose is to estimate the corresponding corrections (*cx*, *cy*), and the lens distortion coefficients (radial distortion coefficients (k1, k2, k3); tangential distortion coefficients (P1, P2); and affinity and non-orthogonality coefficients (B1, B2)). *X_c_* and *Y_c_* represent the calibration parameter for the *x*-axis, R is the radial distance from the origin (principal point) to the projected point in the normalized image plane, and w and h are the image height and width in pixels. The equations are as follows:

(1) $$X_{0}=\frac{X}{Z}\cdot Y_{0}=\frac{Y}{Z}$$

(2) $$R=\sqrt{X_{0}^{2}+Y_{0}^{2}}$$

(3) $$x^{\prime \prime }=\frac{X_{C}}{R}\arctan\left(R\right)$$

(4) $$y^{\prime \prime }=\frac{Y_{C}}{R}\arctan\left(R\right)$$

(5) $$\Delta x=x^{\prime \prime }\left(1+K_{1}r^{2}+K_{2}r^{4}+K_{3}r^{6}+K_{4}r^{8}\right)+\left(P_{1}\left(r^{2}+2x^{\prime \prime 2}\right)+2P_{2}x^{\prime \prime }y^{\prime \prime }\right)$$

(6) $$\Delta y=y^{\prime \prime }\left(1+K_{1}r^{2}+K_{2}r^{4}+K_{3}r^{6}+K_{4}r^{8}\right)+\left(P_{2}\left(r^{2}+2y^{\prime \prime 2}\right)+2P_{1}x^{\prime \prime }y^{\prime \prime }\right)+\;\Delta xB_{1}+\Delta yB_{2}$$

(7) $$x^{\prime }=x_{0}+\;\Delta x+fx^{\prime \prime }+\frac{w}{2}+cx$$

(8) $$y^{\prime }=y_{0}+\;\Delta y+fy^{\prime \prime }+\frac{h}{2}+cy$$

The transformation of 3D points from the local camera coordinate system (*X*, *Y*, *Z*) to 2D projected coordinates (*x*_0_, *y*_0_) in the image coordinate system is governed by the mentioned parameters. The calibrated point coordinates in the image coordinate system are denoted as *x*’ and *y*’, reflecting adjustments made to account (Δx,Δy) for these distortions. In the case of undistorting fisheye images from the corresponding distortions, OpenCV libraries used the inverse mapping of back-projection to the normalized camera coordinate system. The background calibration methods in this research are based on the two main solutions for optic camera calibration: pre-calibration and self-calibration methods, as they are widely implemented and suggested for the case of fisheye calibration (Table 2).

#### 4.2.1. Pre-Calibration

The pre-calibration or chessboard camera calibration method, known as Zhang’s method, involves implementing 2D chessboard images in varied multiple orientations in order to define the camera’s geometric and lens distortion model by calculating the camera calibration parameters [29]. Multiple captures of a known 3D pattern structure beside the unknown position and orientation of each view in 3D space are performed [30]. In this solution, the intrinsic parameters are assumed to be constant during the relative orientation. In other words, extrinsic parameters vary for each image in a relative orientation. Therefore, the re-projection error is minimized between the observed and predicted locations of the square corners, using a nonlinear optimization algorithm [31]. The accuracy and applicability of the imported calibration parameters depends on the quality of the source project photos, the camera stability, and the distance to the objects of interest [30]. In this test, based on the set of images taken from the checkerboard, the positions of the 2D corner points are analysed. These points and the extracted corresponding positions in all images, are used to estimate the distortion of the images and calibration parameters based on the Brown model (Equations (1)–(8)). For the purpose of pre-calibration and network design tests, we captured fisheye images based on the network design configuration in Figure 3, and this configuration of image capturing was repeated for each front and rear lens of the camera individually, and also for each network design test to evaluate the results in Section 5. This solution of camera calibration is also performed by the App Calibrator plugin of MATLAB software.

#### 4.2.2. Self-Calibration

The process of camera calibration, which is performed simultaneously with object identification during photogrammetric evaluation, is referred to as the self-calibration workflow. It makes no assumptions about the 3D structure, using multiple views of an arbitrary scene and rigid structures [30]. The images were automatically matched using the structure from a motion algorithm and the calibration parameters were estimated through the photogrammetric robust optimization of bundle adjustment during self-calibration [28,32]. Since the solution uses the main 3D reconstruction dataset, after the 3D reconstruction in Agisoft Metashape, we can export the self-calibration parameters as well. According to a previous work on calibration analysis [17], the best possible results achieved from the self-calibration parameters were obtained, and these results are used as the reference criteria for the evaluation of other calibration solution results.

#### 4.2.3. Two-Step Calibration

This approach was recently investigated [15,33] in two solutions, but in this research, we improved the pre-calibration stage by using network design tests; so, the best possible pre-calibration parameters were accomplished in this approach:

- Solution 1: Using the best possible results of pre-calibration as initial values of self-calibration.
- Solution 2: Using the best possible results of pre-calibration as calibration parameters to undistort fisheye images, then calculate self-calibration parameters using undistorted images.

**Figure 8:**
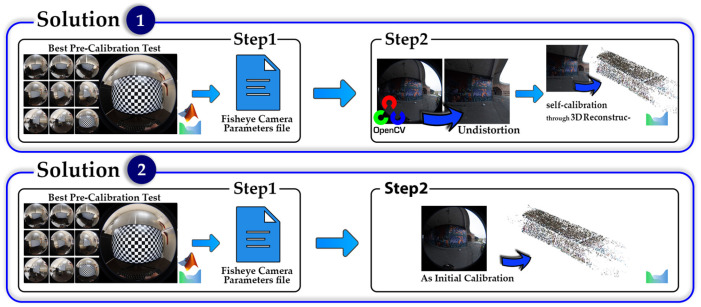
Workflow of two-step calibration procedure.

Solution 1 of the two-step calibration process utilizes the pre-calibration result, specifically, the best set of parameters identified through the network design test, as initial values for conducting the self-calibration test. In contrast, solution 2 follows a different approach; it first applies the best pre-calibration parameters to undistort the fisheye images before using these corrected images in the self-calibration test. In other words, the key distinction between the two solutions lies in whether or not the undistortion process is applied. For solution 1, in the first step, we consider the main workflow of pre-calibration, which includes the multiple images captured from the 2D chessboard to estimate calibration parameters as well. The best camera configuration strategy used for the imagery procedure of pre-calibration is based on the results of the network design scenario assessment (Figure 3). In the next step, all 10 parameters of calibration obtained from the pre-calibration method are used as the initial values for the self-calibration method. Thus, the initial values of image distortion parameters in the self-calibration method will be closer to the correct values compared to the scenario where their initial value is assumed to be zero. Moreover, all ten calibration parameters will be estimated in the relative orientation calculations and 3D reconstruction. Alongside the estimated calibration parameters, the 3D reconstructed points will also be obtained, which will be explained in Section 4.3. The results of the 3D reconstruction and calibration using the two-step calibration method will be presented in Figure 8. For solution 2, same workflow occurs until the pre-calibration is achieved. In order to undistort underpass images, OpenCV computer vision libraries of fisheye geometry were used, and in the final step, undistorted images were imported in Agisoft Metashape, but with the frame sensor type definition included, and then the procedure was repeated by self-calibration to achieve the final calibration results (Figure 8).

#### 4.2.4. 3D Calibration

**Figure 9:**
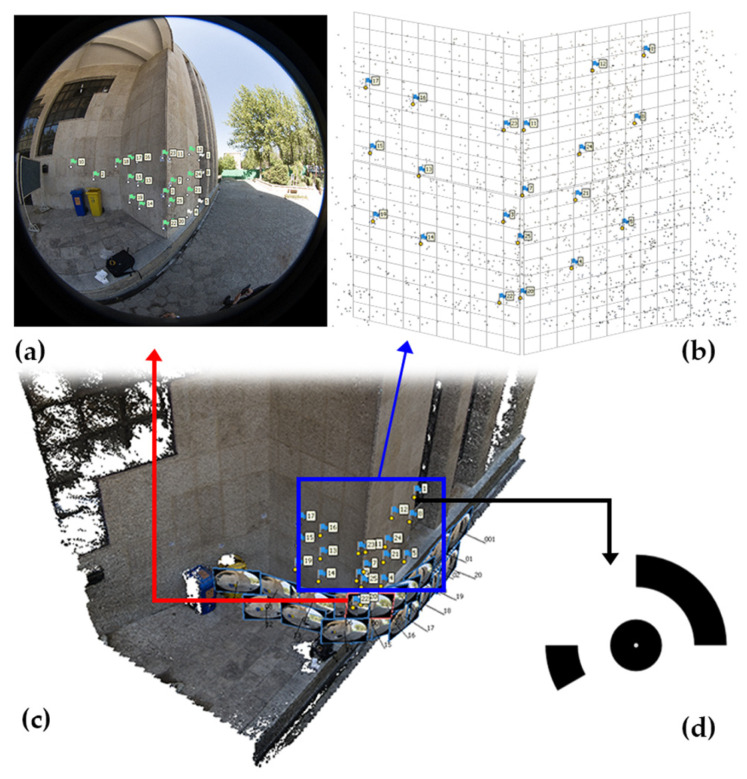
3D camera calibration test: (**a**) 25 targets attached on the surface of the outer corner of the walls. (**b**) Marker detection manually on each image. (**c**) Network of the observed control points. (**d**) Shape of the attached marker signs.

To improve the accuracy of the self-calibration method, the bundle adjustment is optimized by incorporating control points from the captured scene. These points are printed on A5-sized pages (21 cm × 14.8 cm), based on coded targets that can be generated by the Agisoft Metashape software. In the 3D calibration method, points are placed on multiple surfaces at different depths and distances from each other; therefore, targets are placed on two walls. The positions of these points were measured and stored based on a local coordinate system using a total station. These coordinates were then imported into the Metashape software as ground control point coordinates. The two-dimensional positions of the images were automatically determined by the software based on the unique configuration of each target relative to each other, and the accuracy of the positioning of the photographic points was manually verified. By performing the relative orientation process and using the control points in the bundle adjustment, the calibration parameters are optimized (Figure 9).

### 4.3. 3D Reconstruction Tests

One of the reasons for the thickness (noise) of the reconstructed surfaces in the point cloud (point cloud noise) and the geometric stability or deformation of the reconstructed structure is the inaccurate calibration parameters used in the 3D reconstruction process. Calibration parameters can be used in producing the 3D point cloud either as fixed values (known as pre-calibration) or as initial values for re-estimating parameters during the bundle adjustment optimization. Since the projection model used in the calibration procedures is a standard package and well-known in photogrammetry fundamentals, it is reasonable to compare the corresponding results and applicability to practical work such as 3D reconstruction. Accordingly, in this test, the impact and accuracy of these two methods will again be assessed concerning the precision and quality of the 3D reconstruction. Considering the principles of network design for imaging with low-cost spherical cameras, a previous work is followed [17]. After the data acquisition step, the images were processed in Metashape software and 3D reconstruction was accomplished through relative orientation and bundle adjustment. The reconstruction process operated on a specific PC (GPU device: NVIDIA GeForce 930Mx-3 compute units @901 MHz, 2047 MB, CUDA). This process was performed once by inputting the calibration parameters from the pre-calibration method (based on the best results obtained from network design tests) as fixed values (Section 4.2.1), ensuring that no updates to the calibration parameters occur during the bundle adjustment optimization. Also, once again, the same initial values of the camera calibration were implemented, and the parameter update was permitted during the optimization (Section 4.2.3: first solution). In the other tests, the calibration parameters derived from the output of the 3D reconstruction were based on the self-calibration test (Section 4.2.3 and Section 4.2.4). Table 3 specifies the optimal settings of the 3D reconstruction for each calibration method used.

The point cloud generation, through the structure from the motion and bundle adjustment [28], which represents the best nonlinear estimator based on the image geometry, starts with importing the underpass images in the workspace of Agisoft Metashape. After the definition of the fisheye sensor type and the initial default setting for relative orientation (Table 3), the calculation of relative orientation begins. The 3D point cloud of the 3D calibration dataset is generated by the manual marker positioning of all 25 control points on the surfaces of two walls. After the initial procedure of bundle adjustment, the estimation of unknown parameters will be updated once again with the constraint of added control points to the bundle adjustment optimizer. The correspondent relative orientation and bundle adjustment settings are the same as mentioned in Table 3.

### 4.4. Quality Analysis

#### 4.4.1. Tie Points Enhancement and Re-Projection Error Analysis

To evaluate the accuracy of the calibration, the RMS residual (the unit is meter based on the global coordinate system) is used in each calibration solution to represent the re-projection errors for the tie points detected on the source images, averaged across all the images of the calibration group. The values corresponding to each item are listed. Moreover, the number of achieved tie points after the stage of alignment, for each calibration test, are demonstrated in Section 5.

#### 4.4.2. Control Points Accuracy Analysis

Similarly, for absolute orientation accuracy, two criteria are considered, including the control point RMS error (or GCPs accuracy) and check point RMS error (or check point accuracy). As well, to compare the quality, at this stage we demonstrated the GCPs accuracy of the results, shown in Figure 13.

#### 4.4.3. Distance Analysis

The corresponding distances of the two point clouds were calculated by the cloud to cloud distance measurement (C2C). The point clouds are imported into the Cloud Compare software (v2.12 beta) and each of them is already georeferenced based on their identical control points. The process starts by selecting one of the point clouds as a reference (LS’s) and then each point of the first point cloud (spherical) is compared to the nearest point in the second point cloud (LS’s) to calculate the required distance.

#### 4.4.4. Geometric Features Analysis

The geometric characteristics of the point cloud were used to assess the geometric consistency and structure of any 3D point cloud in comparison to the associated reference cloud. These are characterized by components of the covariance tensor ($\Sigma_{i}$) [34].

(9) $$\Sigma_{i}=\frac{1}{N}\sum_{n\in P_{i}^{N}}\left(p_{i}-\bar{P}\right){\left(p_{i}-\bar{P}\right)}^{T}$$

where *N* is the number of adjacent points of $p_{i}$ and $\bar{P}$ is the centre of the adjacent. These elements consist of eigenvectors ($e_{1}$, $e_{2}$, $e_{3}$) and eigenvalues ($\lambda_{1}$, $\lambda_{2}$, $\lambda_{3}$) of the covariance tensor, and the combination of these parameters leads to the definition of a unique geometric feature of the point clouds, all of which use sensors that are imported separately in Cloud Compare. These features included surface density, verticality, planarity, and roughness, defined within a neighborhood with respect to each point, which relies on the sampling distance. The radius (*r*) for neighboring points ($p_{i}$), the mean plane or surface (μ), and the number of points in the specific radius (N) can be determined in the Cloud Compare toolbox, and the following results can show their present effect on spherical point clouds and the proper geometric structure on the laser scanner reference as well. The following equations define each of the geometric features for the assessment of the structure of the spherical point cloud (Equations (10)–(13)) [35,36].

(10) $$\mathrm{VERTICALITY}:\;1-p_{i}$$

(11) $$\mathrm{ROUGHNESS}:\;p_{i}-\mu$$

(12) $$\mathrm{PLANARITY:}\;\frac{\lambda_{2}-\lambda_{3}}{\lambda_{1}}$$

(13) $$\mathrm{DENSITY:}\;\frac{N}{\left(\frac{4}{3}\times \pi \times r^{3}\right)}$$

## 5. Results

This research aimed to analyse the impact of different camera calibration solutions on the resulting 3D point cloud. To assess the results, we divide the evaluation stages into absolute and relative sections. First, the accuracy and correctness of the calibration parameters in each pre-calibration test are evaluated using the mean and standard deviation as key statistical measures. Based on the results, the best calibration method for the network design test is identified. Meanwhile, we also compare the software’s performance. After selecting the best pre-calibration test, we proceed to the relative evaluation section. In this phase, we compare the results of four calibration methods based on three main parameters related to the accuracy and consistency of the calibration tests. These parameters included factors such as noise, geometric consistency, completeness based on re-projection error, number of tie points, and control point accuracy, which are derived using calibration parameters in the 3D reconstruction of the underpass. Additionally, we assess and compare the geometric features of the generated 3D point clouds (produced by each calibration method) and their alignment accuracy with respect to the LS point cloud.

### 5.1. Absolute Evaluation

**Figure 10:**
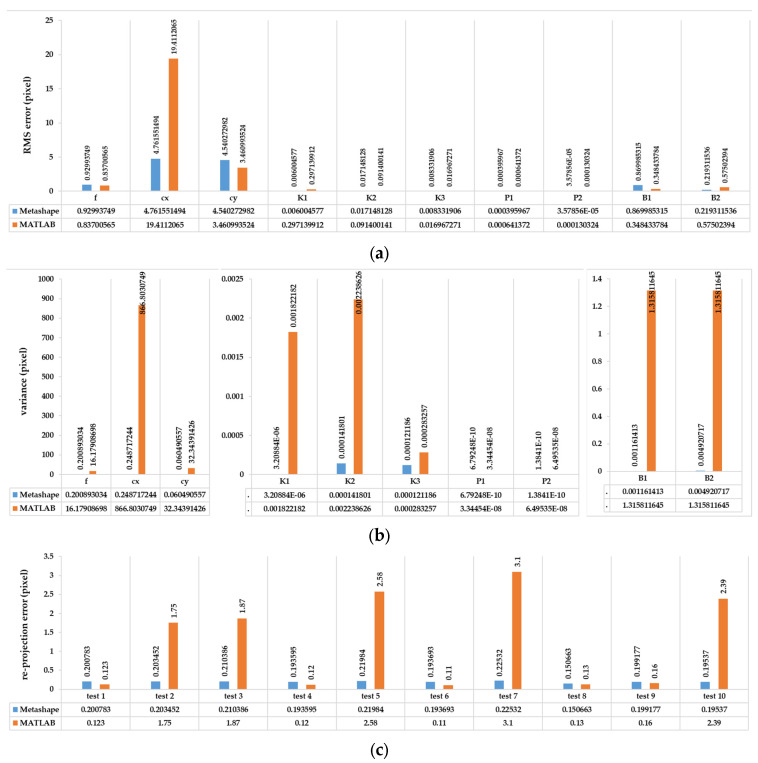
The performance comparison of front lens of Insta360 One X2 pre-calibration and network design, between Agisoft Metashape and MATLAB in terms of (**a**) RMSE error and (**b**) variance of calibration parameters for all ten tests; also, (**c**) shows the re-projection error of each network design test for the estimation of calibration parameters. The results of (**c**) also illustrate the consistency and accuracy of fisheye calibration parameters for the pre-calibration test.

**Figure 11:**
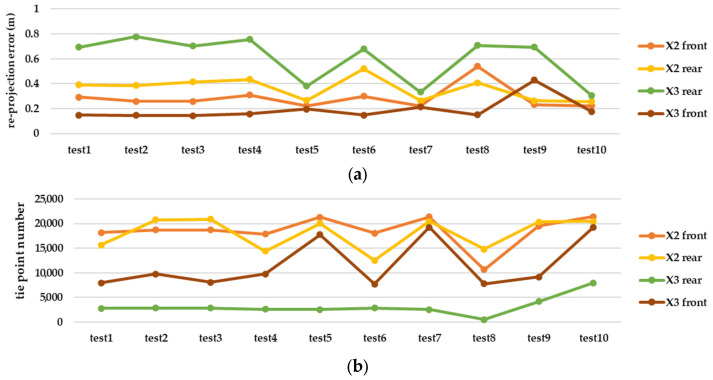
The effect of each calibration parameter from each pre-calibration test in the terms of (**a**) re-projection error of each camera and each lens and (**b**) tie point number of each camera and each lens, on 3D reconstruction of graffiti underpass.

**Figure 12:**
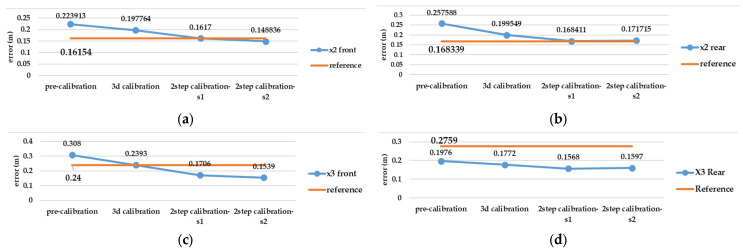
Evaluation calibration tests result according to re-projection error criteria in Agisoft Metashape (unit: relative coordinate system (m)): re-projection error in 3D space for (**a**) front lens and (**b**) rear lens of Insta360 X2, and (**c**) front lens and (**d**) rear lens of Insta360 X3.

**Figure 13:**
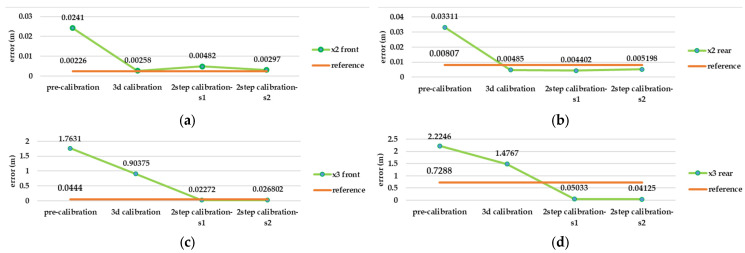
Evaluation calibration tests result according to control point error criteria in Agisoft Metashape (unit: meter): control point error in 3D space for (**a**) front lens and (**b**) rear lens of Insta360 X2, and (**c**) front lens and (**d**) rear lens of Insta360 X3.

**Figure 14:**
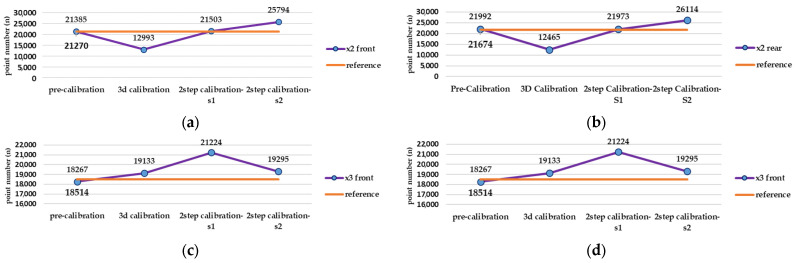
Evaluation calibration tests result according to tie point number enhancement criteria in Agisoft Metashape (unit: points): number of tie point after alignment process in (**a**) front lens and (**b**) rear lens of Insta360 X2, and (**c**) front lens and (**d**) rear lens of Insta360 X3.

**Figure 15:**
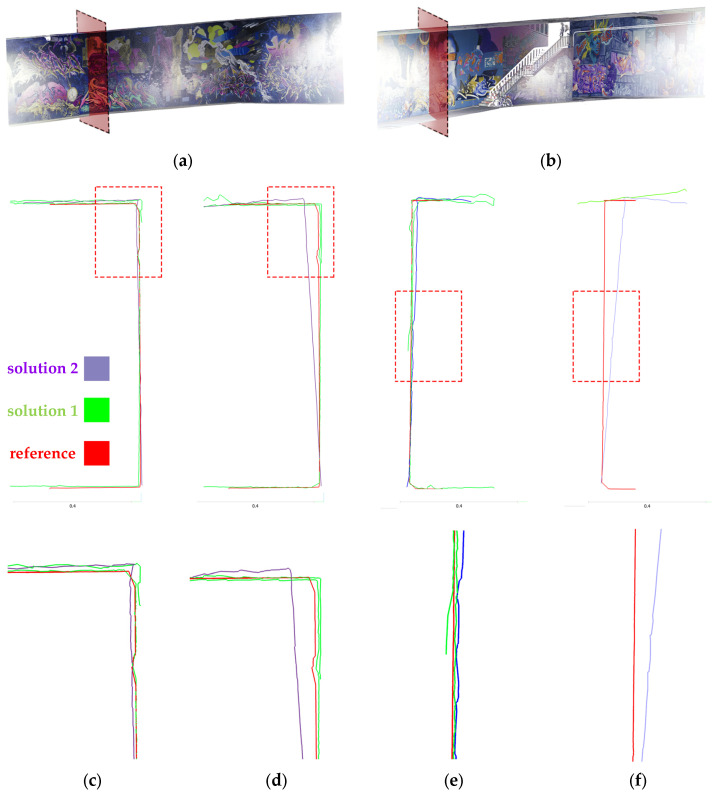
The 2D profiles of (**a**) front and (**b**) rear side of the underpass tunnel: 3D point cloud of (**c**) X2-front lens, (**d**) X3-front lens, (**e**) X2-rear lens, and (**f**) X3-rear lens.

In the absolute evaluation of calibration parameters, the standard deviations and mean differences of the calibration parameters compared and analysed via the reference method. The reference method was chosen based on results from a previous study [17] and also other studies [37] utilizing self-calibration. Among the ten tests performed using the pre-calibration method (Figure 3, Section 4.1 and Section 4.2.1), test number 10 was identified as the best in terms of the accuracy and reliability of the estimated parameters (based on Figure 10c and Figure 11). This test, in terms of network design, involved altering the image depth and increasing the number of images, compared to the initial base test with nine images. Therefore, the result of test number 10 was considered for the main pre-calibration test in the following report (Figure 10, Figure 11, Figure 12, Figure 13, Figure 14 and Figure 15). The RMS error for the estimated calibration parameters for the front and rear lenses of the X2 camera was 2.18 and 3.379 pixels, and for the X3 camera, it was 39.79 and 40.80 pixels, respectively.

Since the front lens of both cameras has slightly better performance in terms of the re-projection error and accuracy of calibration parameters (Figure 10, Figure 11 and Figure 12), the performance evaluation between MATLAB and Metashape in terms of pre-calibration accuracy, consistency, and correctness has been illustrated by the front lens, and only by using the Insta360 One X2 camera. These programs utilize the Brown mathematical model to address the geometry and distortion of fisheye images [28,38]. The same procedure for the pre-calibration tests and network design in MATLAB software was repeated, and the result showed a weak ability in the consistency and accuracy of the calibration parameters in MATLAB (Figure 10), especially for estimating the pixel position of the geometric centre of the image and focal length. Also, there was much more incorrect positioning of the camera locations after calibration.

Regarding the large error in the results of the camera calibration in MATLAB software, the rest of the pre-calibration tests were repeated with another lens and camera in Agisoft Metashape. As shown in Figure 10, Metashape has dramatically better performance in each of the three stages of consistency, accuracy, and reliability. Radial and tangential distortion coefficients have strong consistency, according to the main parameters (focal length and geometric centre of the image) among the test results. Considering the impact of each calibration parameter from each test on the 3D reconstruction (Figure 11), the best result was obtained for Insta360 One X2, despite the close similarity with Insta360 One X3. Given the results, which include a 0.223 and 0.308 re-projection error and 21,385 and 18,267 tie points, the accuracy is better compared to the rear lens in X2 and X3, respectively.

The number of correct camera positionings are demonstrated in Table 4. The missed alignment of the test is the aggregation of previous tests for the inefficient camera positions during image capturing. However, the results show five camera misalignments and seven incorrect positionings for the rear, and, respectively, four and five values for the rear lens, only for the pre-calibration test (test number 10). Other calibration solutions performed perfectly while using both spherical cameras and their correspondent lenses (Table 4).

### 5.2. Relative Evaluation

Based on the best possible network design in test number 10, there is remarkable gap between the pre-calibration and the reference calibration solution (self-calibration) in terms of accuracy and completeness of the point cloud (according to Figure 12, Figure 13 and Figure 14), but other strategies such as 3D calibration and two-step calibration confirmed the close accuracy and were even better than the reference solution. In addition to the accuracy and completeness of the generated point cloud, the images used in the second solution of the two-step calibration method were exported as undistorted images using OpenCV libraries. The second solution of the two-step calibration has the best performance in terms of accuracy and completeness and was also better than the self-calibration stage. The mean variance of the estimated parameters in the first and second solutions of the two-step calibration methods were 3.28 and 4.80, respectively, indicating the stability of the parameters in these methods as well. The average improvement of the re-projection error is 0.61 among all the lenses and was compared to the self-calibration solution. The second solution in the two-step calibration also had better results in terms of tie points and control point errors, with a mean improvement of 541 points and 0.17 m. On the other hand, the 3D calibration had the closest behaviour to the reference, which may be a consequence of these two calibrations having more similar calculations and procedures. The first solution of the two-step calibration had a remarkable accuracy and was almost more accurate than the 3D calibration (Figure 12, Figure 13 and Figure 14).

Test number 10, among those performed in the pre-calibration phase, included both the criteria of varying the depth of imaging and increasing the number of images taken. Consequently, it can be concluded that capturing as many angles as possible ensures a complete visibility of the checkerboard pattern and its points and can lead to a higher accuracy and reliability in the parameter estimation, thus improving the quality of the 3D reconstruction. The re-projection error of test number 10 indicates improvements of 0.07 and 0.14 for the front and rear lens, compared to the initial imaging state with only nine images in a single run (test number 1, Figure 3). According to the variance of pre-calibration parameters, the maximum observed variance pertained to the estimation of the focal length and the x-component of the geometric centre displacement, with a relative stability in estimating other calibration parameters across the conducted tests (Figure 10b,c). It also demonstrates acceptable stability across the ten tests conducted after each camera use for imaging and processing, which was expected to be unstable for any non-metric camera (Figure 10b). The results of Agisoft Metashape seems far more accurate than the MATLAB App calibrator, which can be observed from the large error in estimating the focal length and projection centre (Figure 10). However, the variance discrepancy in MATLAB outputs was significantly higher, indicating less overall stability among the parameters in this software. This behaviour is further confirmed by comparing the RMS error and re-projection of these parameters, suggesting that the parameters estimated with Metashape software exhibit greater accuracy and reliability. One reason for this performance improvement is the exclusion of certain images from calculations due to unsuitable image geometry and inappropriate density of key points, which led to errors in estimating calibration parameters. Thus, despite the inability to estimate the camera’s position and points from all captured images (based on Table 4), Metashape provides higher quality outputs in the pre-calibration method. According to the results of the pre-calibration tests and previous work, this improvement in the estimation of calibration parameters is related to the optimal network design. Nevertheless, the self-calibration method outperformed the pre-calibration method, and the results of the 3D calibration and two-step calibration methods changed the situation. As shown in Figure 13, a slight difference in the re-projection error was observed between the 3D calibration method and the self-calibration method, which also exists among the individual parameters of both methods. Overall, this method can be considered a viable alternative to self-calibration, providing comparable accuracy and precision. Due to the requirements of installing targets at the primary stage for 3D reconstruction, the 3D calibration test was more difficult and time-consuming compared to others. In contrast, the self-calibration method can estimate calibration parameters without targets, relying only on the extracted feature points and the matching process by 3D reconstruction. For a more comprehensive analysis of the results, two sample locations of the final 3D point clouds with each calibration test were compared in terms of surface density, roughness, verticality, and planarity.

In the geometric feature step of this analysis, we can still see the dominant performance of the two-step calibration solutions compared to others. The pre-calibration solution almost had the worst result for each geometric feature. The surface density of the second solution of the two-step calibration is higher than any other existing solution, despite using the same procedure for 3D reconstruction as other calibration results. The outcome of the two-step calibration seems to be remarkable in this test; although the self-calibration is still almost equal, especially when using X2 camera, it even performed better than the two-step calibration. Based on the standard deviation results, the two-step calibration has the most reliable geometric features among the other calibration strategies (Table 5). In order to observe the detailed deviation of the two best solutions for calibration (two-step calibration: solution 1 and 2) between the spherical camera point cloud and the LS (reference), a profile of the front and rear side of the tunnel was extracted from all these three point clouds. It can be claimed that solution 1 contains more noise and causes a duality layer, especially in the corner sides, but in the rear lens model of both cameras, there is only a part of the walls extracted and the lack of completeness is obvious. On the other hand, solution 2 has completeness for the whole wall. If we look at the control points that were taken only on the ground, we can see that even the deviation of the upper side of the spherical point cloud with respect to the LS’s can be justified and distinguished from its geometric defects. The accuracy of the geometric structure of the 3D point cloud is also mentioned in Table 5. This was accomplished by using the 2D profile of specific section of the underpass, in order to clarify the overlapping and the accuracy of the straight walls and flat ceiling (Figure 15).

Our analysis workflow ends with the correctness evaluation of each resulting point cloud. Based on the C2C calculation between each calibration test 3D point cloud and LS point cloud, the best result still explains the advantages of the second solution of the two-step calibration in comparison to others (bold values in Table 6 and Table 7). The front lenses have a lower mean and standard deviation (std) distance compared to the rear lenses.

In the two-step calibration method, the first and second solutions demonstrate improved performance in producing tie points. The increase in the number of tie points and the slight reduction in the re-projection error reflect a decrease in point cloud noise and an increase in point density in the main sections of the captured images. On the other hand, this method ultimately allows for undistorted images to be used in 3D reconstruction without initial calibration parameters. This capability facilitates their processing in many accessible software and toolboxes that exclusively utilize frame images. Based on the accuracy and reliability results of parameters across all conducted tests and prior work, the accuracy of the front lens in estimating calibration parameters and 3D reconstruction is better than the rear lens. Therefore, priority should be given to using front lenses to cover critical areas of the structure or object. In summary, the use of the second solution of the two-step calibration method, through the production of undistorted images based on the calibration parameters from the pre-calibration method (with optimal network design) and the updating of image parameters in 3D reconstruction process, enables better accuracy and point cloud generation from this camera and its fisheye images.

## 6. Discussion

This research aims to provide insights that will refine calibration techniques for spherical cameras based on fisheye projection, ultimately improving the results obtained according to their application in various photogrammetric steps. The results will contribute to a deeper understanding of the calibration process and its impact on the overall quality of photogrammetric data, fostering advancements in the field of spherical and fisheye photogrammetry. The main stage of the calibration strategies involves chessboard pattern imaging, unless the self-calibration technique is used. Therefore, this stage can be critical for any research to evaluate or develop a camera calibration method. Despite the fact that this research was conducted by using various camera calibration methods, there are other factors that can be considered for the distortions of the lens or images, and they need to be modelled and removed from the source dataset. The reference mapping function, imperfections in the manufacturing process of the lens [14], the illumination of the scene, and the calculation algorithm are other factors that can affect the calibration results. The testing workflow and the numerical analyses of the calibration results using different approaches, including the statistical analyses, are other factors to be considered; moreover, the estimation of the accuracy potential in the object space, which was applied using control point measurements, or the relative coordinate systems and re-projection error values, may also affect the calibration results.

## 7. Conclusions and Future Works

In the first step of this research, the best solution to achieve the best possible result of pre-calibration was clarified through various comprehensive network design tests. This involved the acquisition of 26 images in three different distances and the consideration of the main rules of close-range photogrammetry; furthermore, software performance was analysed, which led to the combination of Metashape as the software for relative orientation and calibration tests, and the OpenCV toolbox for undistorted fisheye images. Among the known strategies for fisheye camera calibration, the two-step calibration was confirmed as a reliable and accurate solution for fisheye camera calibration based on the metric criteria such as re-projection error, geometric features, ground control point accuracy, and correctness (due to the C2C distance analysis). Also, if we consider the second solution, which includes an undistortion step, we can claim that the calibration is also applied to the raw dataset of the project in addition to the 3D reconstruction. Despite these advantages, the completeness of the final result still needs to be improved in comparison to the self-calibration solution and the reference 3D model.

Based on recent studies utilizing neural networks and deep learning models for calibrating fisheye images [39,40,41], attention to these methods for achieving better accuracy than the self-calibration method and their encompassing strategies could represent a significant step in enhancing the accuracy and efficiency of optical cameras and the metric products. Additionally, considering the raw data imaging system of these cameras, the common equirectangular format or panorama images can also be accurately calibrated and utilized in 3D reconstruction through a projection system that converts raw images into panorama images and correspondently provides 3D reconstruction.

## Figures and Tables

**Table 1:** Detailed specifications of laser scanner and the correspondent dataset.

Laser Scanner Accuracy Settings	Values
3D Point Accuracy	1.9 mm in 10 m|2.9 mm in 20 m|5.3 mm in 40 m
Ground/Space Sampling Resolution	3 mm (Highest Sampling Resolution)
Angular Accuracy of Ls	18″
Range Accuracy	1 mm in 10 ppm
Optical Camera for Colorized Point Cloud	3 Cameras, 36 Mp Resolution
Noise Distance	0.4 mm in 10 m|0.5 mm in 20 m

**Table 2:** Conclusion of the definition of the calibration strategies; we can summarize the methods, workflows, and considerations in the following table.

Strategies	Workflow	Software/Tools
Self-Calibration	Compute Parameters During 3D Reconstruction	Metashape
Pre-Calibration	Evaluate 10 Individual Pre-Calibration Tests Based On 10 Solutions for Camera Network Design	MATLAB/Metashape
3D Calibration	Capture Test Fields Walls and Use the Targets as Control Point to Achieve Calibration Parameters	Metashape
Two-step Calibration (Solution 1)	Use The Best Pre-Calibration Test Results as Initial Value to Compute the Calibration Parameters Using Self-Calibration Solution	Metashape
Two-step Calibration (Solution 2)	Use The Best Pre-Calibration Test Results to Undistort the Dataset (Fisheye to Normal) and Then Compute the Calibration Parameters Using Self-Calibration Solution	OpenCV/Metashape

**Table 3:** 3D reconstruction alignment settings in Agisoft Metashape software. “Fisheye” represents the fisheye main dataset images and “Normal” represents the undistorted fisheye images.

Alignment Settings	Case Study
Tie Point Threshold	4000 Points
Key Point Threshold	40,000 Points
Sensor Type	Fisheye/Normal
Accuracy Mode	High|Generic Preselection

**Table 4:** Number of incorrect positionings and missed alignments through camera calibration tests in Metashape software.

Error	Sensor	Pre-Calibration	3D Calibration	Two-Step Calibration (Sol1)	Two-Step Calibration (Sol2)	Self-Calibration
**Incorrect Positioning**	**X2**	5	0	0	0	0
**Missed Alignment**		7	0	0	0	0
**Incorrect Positioning**	**X3**	4	0	0	0	0
**Missed Alignment**		5	0	0	0	0

**Table 5:** Based on two sample places of each 3D point cloud from (a) front side and (b) rear side of the underpass, their correspondent geometric features were calculated, the bold numbers indicate the best result.

Calibration	Sensor	Verticality		Planarity		Surface Density		Roughness	
		Mean	Std	Mean	Std	Mean	Std	Mean	Std
**3D Calib**	**X2**	0.9592	0.05109	0.88539	0.0959	16,446	1253	0.0019	0.0022
**Self-Calib**		**0.9605**	0.05206	**0.88837**	0.09689	15,971	1297	**0.0018**	0.0021
**Two-step Calib** (**Sol1**)		0.9592	0.0528	0.8859	**0.09469**	16,437	**1247**	0.0019	0.0022
**Two-step Calib** (**Sol2**)		0.9522	**0.0477**	0.8959	0.1255	**16,909**	1314	0.0019	**0.0020**
**Pre-Calib**		0.3951	0.0597	0.8772	0.11	16,321	1389	0.0021	0.0025
**3D Calib**	**X3**	0.9592	0.05109	0.88539	0.1879	16,446	1253	0.0030	0.004
**Self-Calib**		0.9494	0.07028	0.85501	0.14242	52,689	7376	0.0026	0.0031
**Two-step Calib** (**Sol1**)		**0.9043**	**0.03085**	**0.6448**	0.1662	58.4536	13.6631	0.0121	0.0201
**Two-step Calib** (**Sol2**)		0.9085	0.05517	0.8356	0.173	**55,038**	**7431**	**0.0025**	**0.0027**
**Pre-Calib**		0.9045	0.07229	0.8556	**0.1419**	52,687	7377	0.0026	0.0031

**Table 6:** Mean and standard deviation (STD) of distance per point (with C2C solution) between each fisheye calibration strategy of Insta360 One X2 point cloud and the reference point cloud (LS), the bold numbers indicate the two-step calibration result in compare the reference (self-calibration).

Calibration	Rear X2		Front X2	
	Mean (M)	Std (M)	Mean (M)	Std (M)
**3DCalib**	0.6567	1.359	0.4429	1.2561
**Pre-Calib**	0.3757	0.4145	0.4764	1.0301
**Two-step Calib** (**Sol1**)	0.646	1.3387	0.4284	1.2036
**Two-step Calib** (**Sol2**)	**0.5141**	**1.185**	**0.3664**	**0.9654**
**Self-Calib**	**0.4697**	**0.911**	0.4443	1.2355

**Table 7:** Mean and standard deviations (STD) of distance per point (with C2C solution) between each fisheye calibration strategy of Insta360 One X3 point cloud and the reference point cloud (LS). the bold numbers indicate the two-step calibration result in compare the reference (self-calibration).

Calibration	Rear X2		Front X2	
	Mean (M)	Std (M)	Mean (M)	Std (M)
**3D Calib**	0.5749	1.3318	1.6567	1.0024
**Pre-Calib**	0.456	0.4851	0.602	0.3254
**Two-step Calib** (**Sol1**)	0.656	0.7621	0.2556	0.6538
**Two-step Calib** (**Sol2**)	**0.2013**	**0.3163**	**0.1397**	**0.4747**
**Self-Calib**	0.4838	0.6359	0.2991	0.5391

## Data Availability

Data are contained within the article.
